# Leptin-Notch axis impairs 5-fluorouracil effects on pancreatic cancer

**DOI:** 10.18632/oncotarget.24435

**Published:** 2018-04-06

**Authors:** Adriana Harbuzariu, Ruben Rene Gonzalez-Perez

**Affiliations:** ^1^ Department of Microbiology, Biochemistry and Immunology, Morehouse School of Medicine, Atlanta, GA 30310, USA

**Keywords:** 5-Fluorouracil, pancreatic cancer, leptin, Notch, cytotoxicity

## Abstract

5-FU chemotherapy is a current strategy to treat pancreatic cancer (PC), but unfortunately chemoresistance is eventually developed in most patients. Obesity is a risk factor for PC that could affect 5-FU effectiveness through the adipokine leptin, which is a known proliferation, survival factor and Notch inducer. We investigated whether leptin signaling affects 5-FU cytotoxicity on PC. To this end, tumorspheres developed from BxPC-3 and MiaPaCa-2 PC cells were treated with 5-FU, leptin, inhibitors for Notch (DAPT) and leptin signaling (IONP-LPrA2) and ATP-binding cassette of proteins (Probenecid). Leptin treatment decreased 5-FU cytotoxicity, and increased cell proliferation, colony forming ability, stem cell, pluripotency, EMT markers, drug efflux proteins (ABCC5, ABCC11) and Notch. In addition, leptin reduced the 5-FU effects on apoptosis by decreasing pro-apoptotic (Bax, Caspase-3 activation and PARP degradation) and increasing anti-apoptotic factors (RIP and Bcl-XL). Leptin's effects on PC tumorspheres treated with 5-FU were reduced by IONP-LPrA2 and were mainly Notch signaling- dependent and more evident in MiaPaCa-2-derived tumorspheres. Present results suggest that leptin could impair 5-FU cytotoxicity and promote chemoresistance. Therefore, targeting the leptin-Notch axis could be a novel way to improve 5-FU therapy for PC patients, especially in obesity context.

## INTRODUCTION

Pancreatic cancer (PC) accounts for about 3% of new cases of cancers in the US. The annual death rate for PC patients has remained stable over the past 10 years, with the 5-year relative survival of 8% among cases diagnosed from 2006-2012, followed through 2013 [1]. It is estimated that PC will become the 3rd cause of cancer-related deaths in 2017 [2]. Traditional treatments including surgery and chemotherapy are still relatively ineffective for PC patients. Unfortunately, PC lacks targeted therapies. Moreover, the development of drug resistance significantly decreases the effectiveness of PC chemotherapy.

5-fluorouracil (5-FU), a pyrimidine analog, is a chemotherapeutic drug used in the treatment of a variety of cancer types, including PC. 5-FU exerts its anti-cancer effects through the inhibition of the enzyme thymidylate synthase and the incorporation of 5-FU metabolites into RNA and DNA. PC patients eventually develop 5-FU chemoresistance, which may be related to deficient drug uptake, drug efflux, decreased apoptosis and PCSC actions [3]. 5-FU pretreated PC cells showed higher levels of pluripotency markers Oct-4 and Nanog and PCSC-like phenotype, including increasing capability to form tumorspheres [4–6].

Several factors and mechanisms have been linked to the development of chemoresistance, including cancer stem cells, survival pathways and expression of ATP-binding cassette proteins [7]. Indeed, increased levels of ABCC5 and ABCC11 have been found to be linked to 5-FU chemoresistance [8–10]. Obesity and leptin levels [11, 12] are associated with increased PC risk [13, 14] and development of chemoresistance [15, 16]. Leptin is a hormone and cytokine synthetized by adipocyte, whose levels are proportional to the total body adipose tissue. Overweight and obese individuals show high levels of leptin in blood. Leptin has absolute affinity for its receptor (OB-R), which is mainly expressed in the hypothalamic neurons, where the binding of leptin to OB-R regulates the caloric intake. OB-R is expressed widely throughout the body, including on cells within the pancreas [17]. Notably, OB-R is expressed higher on tumor cells, where its binding to leptin induces cancer cell proliferation, migration, angiogenesis, and reduced apoptosis [18–21]. Higher levels of leptin have been associated with an elevated risk of PC development among men [22]. We previously found that PC cells express OB-R and secrete leptin, suggesting that autocrine leptin signaling might impact on PC development and change responses to chemotherapeutic treatment. Additionally, we have demonstrated that leptin induces Notch expression in PC that was essential for cell proliferation and expansion of PC stem cells (PCSC) [23]. PCSC, identified by CD24^+^CD44^+^ESA^+^, have the capacity to self-renew and lead to the maintenance of the tumor mass. Importantly, PCSC are believed to be involved in the development of drug resistance [24, 25].

Leptin-induced Notch axis in PC was linked to leptin-induced tumor growth [23]. The Notch family consists of four receptors (Notch1-4) that have five ligands named Jagged 1(JAG1), JAG2, Delta-like 1(DLL1), DLL3 and DLL4 [21]. Notch signaling influences cell proliferation, differentiation, apoptosis, cancer cell invasion and metastasis [26]. Additionally, Notch signaling is involved in the maintenance and expansion of cancer stem cells [27]. Several studies have demonstrated that Notch signaling pathway plays an important role in controlling PCSC fate [28]. Over-expression of DLL4 in PC cells stimulates the expression of pluripotency cell markers (Oct-4 and Nanog), resulting in increased number of PCSC [29, 30]. Stem cells positive for Sox-2, Oct-4 and Nanog show higher aggressive growth, invasion, migration potential, and enhanced drug resistance properties [31]. PCSC show considerably higher levels of Notch1 and Notch2 than non-malignant pancreatic stem cells [32, 33]. Additionally, the activation of Notch3 in PC was associated with more aggressive tumors. Notch3 and Hey-1 expression was associated with reduced overall and disease-free survival following tumor resection [34]. Furthermore, PC cells upregulate Notch4 that was linked to chemotherapy resistance. Indeed, downregulation of Notch4 using siRNA sensitized PC cells to docetaxel [35].

It is known that leptin can interfere with 5-FU cytotoxic effects on colon cancer cells and stem cells [36, 37]. However, it is unknown whether leptin impairs 5-FU actions on PC. To this end, the impact of leptin signaling on 5-FU cytotoxic and pro-apoptotic effects were investigated in tumorspheres from two PC cell lines. Additionally, it was determined whether leptin signaling regulates ATP-binding cassette proteins in PC treated with 5-FU and whether leptin-induced Notch is involved in 5-FU drug resistance. Present results suggest that leptin could interfere with 5-FU effectiveness on PC. Leptin could be an important endogenous survival and pro-chemoresistance factor for PC patients treated with 5-FU. Mainly, the effects of leptin were related to increased PC proliferation, maintenance of PCSC, expression of pluripotency, EMT factors, and expression of ABCC5 and ABCC11 drug efflux-proteins, which were generally linked to leptin-induced activation of Notch. Overall, it was found that 5-FU kills PC cells but spares PCSC, which were further rescued by leptin. Leptin is likely an important survival factor for PC that significantly impairs the effectiveness of 5-FU.

## RESULTS

### Leptin induces cell proliferation and colony forming ability in 5-FU treated PC tumorspheres

**Figure 1:**
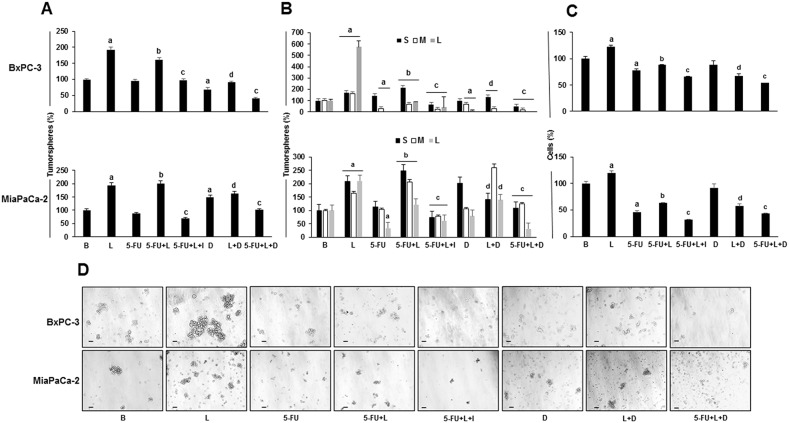
Leptin increases the number and size of 5-FU treated tumorspheres **(A)** Tumorsphere number; **(B)** Tumorsphere size; **(C)** Number of cells in tumorspheres; **(D)** Representative images of tumorspheres after treatments (bar = 60 μm). PC cells were cultured (20,000 cells/well) in 6 wells low attachment plates in Mammocult medium containing 5-FU (20 μg/ml), leptin (L; 1.2nM), IONP-LPrA2 (I; 0.0036 pM) and DAPT (D, γ-secretase inhibitor; 20 μM) for one week. Tumorspheres were dissociated by mechanical means and cell number was determined. Untreated tumorspheres were used as control (Basal, B). Effects of treatments on tumorspheres and cell number were expressed as % of control. Experiments were repeated three times. a: p≤0.05 compared to B; b: p≤0.05 compared to 5-FU; c: p≤0.05 compared to 5-FU+L; d: p≤0.05 compared to L.

To investigate the potential role of leptin on reducing the 5-FU cytotoxic effect on PC growth and aggressiveness, tumorspheres from BxPC-3 and MiaPaCa-2 cells were treated for 7 days with 5-FU and leptin (Figure 1A–1D). Leptin increased tumorsphere number and size, and the number of cells forming tumorspheres in both cell lines (Figure 1A–1C). 5-FU did not affect the tumorsphere number but reduced their size (medium and large) in BxPC-3, the less aggressive cell line (Figure 1A and 1B). However, in the more aggressive cell line MiaPaCa-2, 5-FU reduced both tumorsphere number and size (large) (Figure 1A and 1B). These 5-FU effects were accompanied by a reduction of cell number composing tumorspheres from both PC cell lines (Figure 1C). The addition of leptin reverted the damage induced by 5-FU on PC tumorspheres (Figure 1A–1C). The use of IONP-LPrA2 further enhanced 5-FU cytotoxic effects by reducing PC tumorsphere size and cell number. The addition of the Notch inhibitor (DAPT) reduced leptin's effects on tumorspheres treated with 5-FU (Figure 1A–1C). This suggests that leptin signaling effects on PC tumorspheres treated with 5-FU were related to the activation of Notch signaling. Data from cell counting show that leptin impacted higher on cell survival in 5-FU treated MiaPaCa-2 tumorspheres compared to those derived from BxPC-3 cells, which was related to Notch signaling (Figure 1C). These results suggest that 5-FU spares chemoresistant cells with active Notch signaling (i.e. PCSC), which were rescued by leptin. It has been shown that PCSC overexpress OB-R [38]. Notch signaling was involved in leptin induced cell proliferation, but it did not affect PC tumorspheres when leptin was not added (Figure 1C).

### Leptin induces Notch+ cells in 5-FU treated PC tumorspheres

**Figure 2:**
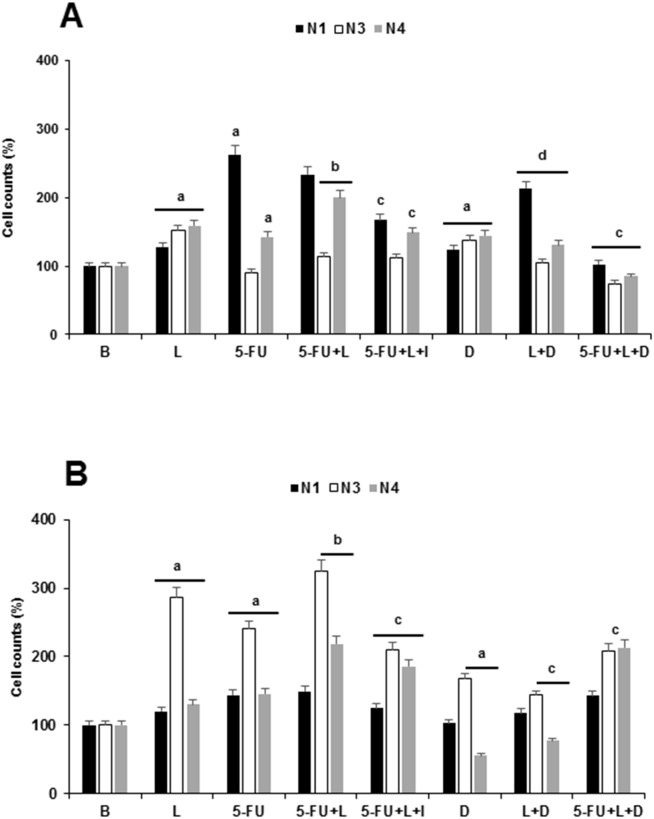
Effects of 5-FU, leptin and DAPT on Notch positive cells in tumorspheres Counts of Notch1^+^, Notch3^+^ and Notch4^+^ cells from **(A)** BxPC-3 and **(B)** MiaPaCa-2 tumorspheres. PC tumorspheres were cultured (20,000 cells/well) in 6 wells low attachment plates in Mammocult medium containing 5-FU (20 μg/ml), leptin (L; 1.2 nM), IONP-LPrA2 (I; 0.0036 pM) and DAPT (D, γ-secretase inhibitor; 20 μM) for one week. Notch^+^ cells were determined by flow cytometry analysis. Untreated cells were used as control (Basal, B). Effects of treatment on number of cells were expressed as % of control. Experiments were repeated three times. a: p≤0.05 compared to B; b: p≤0.05 compared to 5-FU; c: p≤0.05 compared to 5-FU+L; d: p≤0.05 compared to L. N1: Notch1; N3: Notch3; N4: Notch4.

Figure 2 shows the effects of 5-FU treatment on the number of Notch+ cells in PC tumorspheres. 5-FU kills PC cells (see Figure 1), but the relative increase of Notch+ cells suggested that 5-FU spared cells that express Notch1 and Notch4 in BxPC-3 tumorspheres (Figure 2A) and Notch 1,3 and 4 in MiaPaCa-2 tumorspheres (Figure 2B). However, 5-FU did not change the levels of Notch expression in PC cells (data not shown). It was assessed that leptin increases PC cells proliferation (see Figure 1), and the number of cells that express Notch3 and Notch4 in tumorspheres from both cell lines treated with 5-FU (Figure 2A and 2B). Leptin's effects were diminished by the addition of leptin antagonist IONP-LPrA2 to 5-FU, except for the number of BxPC-3 Notch3^+^ cells (Figure 2A and 2B). When DAPT (γ-secretase inhibitor) was added to PC tumorspheres treated with 5-FU and leptin, the number of BxPC-3 Notch3 and Notch4 positive cells decreased, while only MiaPaCa-2 Notch3^+^ cells were reduced. (Figure 2A and 2B). These results suggest that the activation of Notch signaling is needed for leptin's effects on the proliferation and survival of Notch^+^ cells in PC tumorspheres treated with 5-FU.

### Leptin increases PCSC, pluripotency and EMT positive cells in 5-FU treated PC tumorspheres

**Figure 3:**
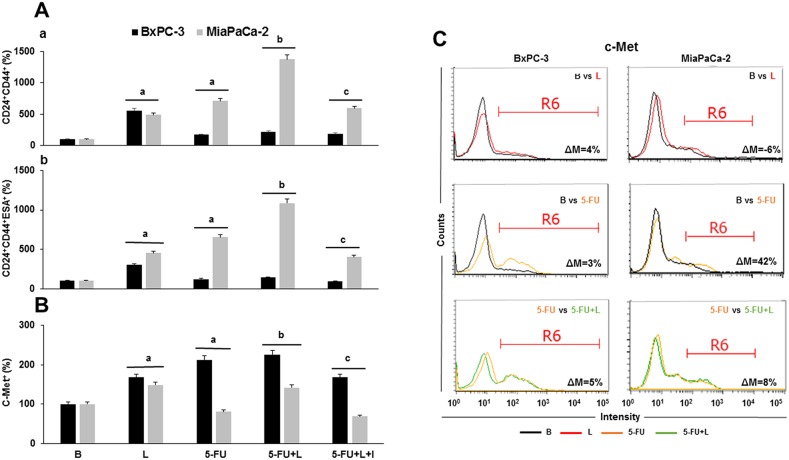
Leptin increases the number of cells expressing stem cell markers in 5-FU treated- tumorspheres **(A)** Percent of cells: (a) CD24^+^CD44^+^ and (b) CD24^+^CD44^+^ESA^+^; **(B)** Percent of c-Met^+^ cells from PC tumorspheres **(C)** Histograms for c-Met expression. c-Met^+^ expression was analyzed as mean fluorescent intensity (MFI%) and the difference between two treatments MFI was calculated (ΔM%). R6 gate was set on positive events in the histograms. Number of CD24^+^CD44^+^ESA^+^ and c-Met^+^ cells as determined by dot plot flow cytometry analysis. Untreated cells were used as negative control (Basal, B). Effects of treatment on tumorspheres and cell number were expressed as % of control. Experiments were performed three times. a: p≤0.05 compared to control; b: p≤0.05 compared to 5-FU; c: p≤0.05 compared to 5-FU+L; ΔM=variation of median fluorescence intensity.

To investigate whether leptin increases stemness and metastasis potential in 5-FU treated PC tumorspheres, the number of PCSC^+^, pluripotency^+^ and EMT^+^ cells was determined. 5-FU killed PC cells, but spared cells expressing PCSC markers. Therefore, the relative number of CD24^+^CD44^+^ and CD24^+^CD44^+^ESA^+^ cells from PC tumorspheres was increased (Figure 3A). Remarkably, leptin increased the proliferation and survival of PC cells from tumorspheres (see Figure 1) and augmented even more the number of PCSC^+^ cells in 5-FU treated PC tumorspheres (Figure 3A). Notably, leptin's effects were more evident in MiaPaCa-2 tumorspheres. Interestingly, the number of MiaPaCa-2 c-Met^+^ cells were decreased by 5-FU, but the addition of leptin augmented the number of these cells to levels higher than in untreated control. Furthermore, flow cytometry analysis showed that 5-FU increased c-Met expression (42% > basal), which was further increased by leptin (8% > 5-FU) (Figure 3C). These leptin's effects were not affected by the addition of DAPT (data not shown), which suggests that c-Met expression was independent of Notch pathway activation in PC tumorspheres.

**Figure 4:**
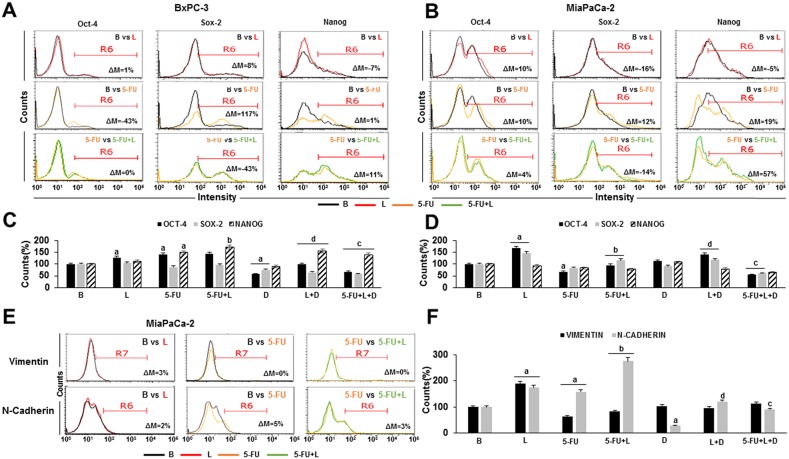
Leptin increases pluripotency and EMT markers expressing cells in 5-FU treated- tumorspheres Flow cytometry histograms for Oct-4, Sox-2, Nanog, Vimentin and N-cadherin expression in BxPC-3 **(A)** and MiaPaCa-2 **(B, E)** tumorsphere-derived cells. Percent of Oct-4^+^, Sox-2^+^, Nanog+ **(C, D)**, Vimentin^+^ and N-cadherin^+^ **(F)** cells in PC tumorspheres. Positive expression of proteins was analyzed as mean fluorescent intensity (MFI%) and the difference between two treatments MFI was calculated (ΔM%). R6 gate was set on positive events in the histograms. The number of positive cells were analyzed using flow cytometry dot plots. Unstained cells were used as negative control. PC tumorspheres were cultured in 6 wells low-attachment plates in Mammocult medium containing 5-FU (20 μg/ml), leptin (L; 1.2 nM), IONP-LPrA2 (I; 0.0036 pM) and DAPT (D, γ-secretase inhibitor; 20 μM) for one week. Untreated PC tumorspheres were used as control (Basal, B). Effects of treatment on tumorspheres and cell number were expressed as % of control. Experiments were performed three times. a: p≤0.05 compared to control; b: p≤0.05 compared to 5-FU; c: p≤0.05 compared to 5-FU+L; d: p≤0.05 compared to L; ΔM=% of median fluorescence changes.

In BxPC-3 tumorspheres cells, 5-FU decreased Oct-4 (43% < basal) and increased Sox-2 (117% > basal), but did not change Nanog expression (Figure 4A). Moreover, 5-FU spared Oct4^+^ and Nanog^+^ BxPC-3 cells (Figure 4C). In contrast, leptin did not affect Oct-4, but decreased Sox-2 (43% < 5-FU) and increased Nanog (11% > 5-FU) expression in BxPC-3 tumorspheres (Figure 4A). Additionally, leptin signaling increased the number of Nanog^+^ BxPC-3 cells in tumorspheres treated with 5-FU (Figure 4C). In MiaPaCa-2 tumorspheres, 5-FU increased the expression of all pluripotency markers (Oct-4 > 10%, Sox-2 > 12%, Nanog > 19% compared to basal) (Figure 4B). Nevertheless, 5-FU treatment did not change the relative number of Sox-2^+^ and Nanog^+^, but reduced Oct-4^+^ MiaPaCa-2 cells (Figure 4D). Leptin increased further Oct-4 (4%) and Nanog (57%) but reduced Sox-2 expression on 5-FU treated MiaPaCa-2 tumorsphere-derived cells (Figure 4B). In addition, leptin also increased the number of Oct-4^+^ and Sox-2^+^ MiaPaCa-2 cells in 5-FU treated tumorspheres (Figure 4D). These leptin's effects were abrogated by DAPT (data not shown).

It was further investigated whether 5-FU and leptin affect EMT markers in PC tumorspheres. The expression levels and number of Vimentin^+^ or N-cadherin^+^ cells from BxPC-3 tumorspheres were not changed by 5-FU or leptin treatments (data not shown). However, 5-FU slightly increased N-cadherin expression (by 5%) (Figure 4E) and spared N-cadherin^+^ cells in MiaPaCa-2 tumorspheres (Figure 4F). Leptin increased the effects of 5-FU on N-cadherin^+^ cells in MiaPaCa-2 tumorspheres, which were inhibited by DAPT (Figure 4F). These results suggest that leptin could increase the aggressiveness and metastatic potential of MiaPaCa-2 by increasing the number of EMT^+^ cells (Vimentin^+^ and N-cadherin^+^), which involved Notch signaling.

### Leptin increases the expression levels and number of ABCC5^+^ and ABCC11^+^ cells in 5-FU treated PC tumorspheres

**Figure 5:**
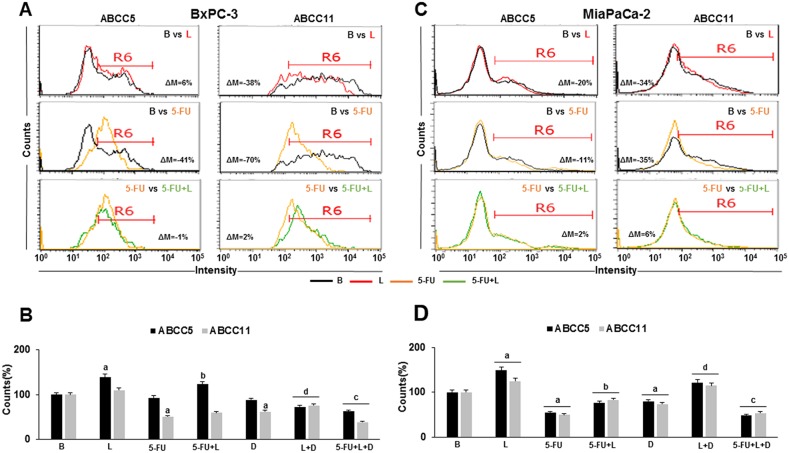
Leptin increases ABCC5 and ABCC11 expressing cells in 5-FU treated- tumorspheres Flow cytometry histograms for ABCC5 and ABCC11 expression in BxPC-3 **(A)** and MiaPaCa-2 **(C)** tumorsphere-derived cells. Percent of ABCC5^+^ and ABCC11^+^ BxPC-3 **(B)** and MiaPaCa-2 **(D)** cells in PC tumorspheres. Positive expression of proteins was analyzed as mean fluorescent intensity (MFI%) and the difference between two treatments MFI was calculated (ΔM%). R6 gate was set on positive events in the histograms. The number of positive cells were analyzed using flow cytometry dot plots. Unstained cells were used as negative control. PC tumorspheres were cultured in 6 wells low-attachment plates in Mammocult medium containing 5-FU (20 μg/ml), leptin (L; 1.2 nM), IONP-LPrA2 (I; 0.0036 pM) and DAPT (D, γ-secretase inhibitor; 20 μM) for one week. The cells positive for ABCC5 and ABCC11 were determined by flow cytometry analysis. Untreated cells were used as control (Basal, B). Effects of treatment on tumorspheres and cell number were expressed as % of control. Experiments were performed three times. a: p≤0.05 compared to control; b: p≤0.05 compared to 5-FU; c: p≤0.05 compared to 5-FU+L; d: p≤0.05 compared to L; ΔM=% of median fluorescence changes.

It was determined whether 5-FU and leptin affect the expression and the number of ABCC5^+^ and ABCC11^+^ cells in PC tumorspheres. 5-FU decreased the expression levels of ABCC5 (41% in BxPC-3 and 11% in MiaPaCa-2) and ABCC11 (70% in BxPC-3 and 35% in MiaPaCa-2) (Figure 5A and Figure 5C). 5-FU also reduced the number of ABCC11^+^ cells in BxPC-3 and both ABCC5^+^ and ABCC11^+^ cells in MiaPaCa-2 tumorspheres (Figure 5B and 5D). Leptin increased the number of ABCC5^+^ and ABCC11^+^ cells in PC tumorspheres from both cell lines (Figure 5B and 5D). Additionally, leptin increased the number of ABCC5^+^ and ABCC11^+^ cells in 5-FU treated MiaPaCa-2 tumorspheres, while only the number of BxPC-3 ABCC5^+^ cells were increased by 5-FU (Figure 5B and 5D). The inhibition of Notch signaling reduced leptin's effects in both cell lines (Figure 5B and 5D). These results suggest that leptin's effects on BxPC-3 and MiaPaCa-2 ABCC^+^ tumorspheres were Notch dependent.

### Leptin decreases 5-FU cytotoxicity on PC tumorspheres through ABCC proteins

**Figure 6:**
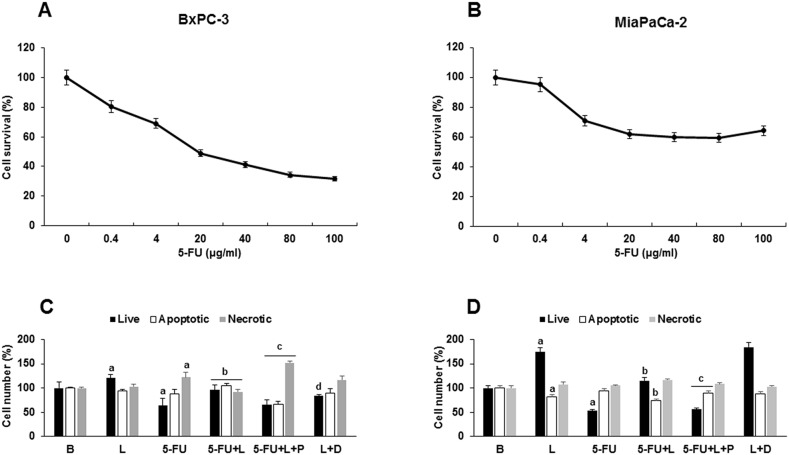
Leptin- induced survival of 5-FU treated- tumorspheres involves ABCC proteins Effects of 5-FU on survival of **(A)** BxPC-3 and **(B)** MiaPaCa-2 tumorspheres. Effects of 5-FU, leptin, DAPT and Probenecid on survival of **(C)** BxPC-3 and **(D)** MiaPaCa-2 cells forming tumorspheres. Cell numbers (live, apoptotic and necrotic) were determined after 1-2 days of treatment. PC tumorspheres were cultured (20,000 cells/well) in 6 wells low attachment plates in Mammocult medium containing 5-FU (20 μg/ml), leptin (L; 1.2 nM), IONP-LPrA2 (I; 0.0036 pM), DAPT (D, γ-secretase inhibitor; 20 μM) and Probenecid (P, ABCC inhibitor; 2 mmol/ml) for one week. Untreated cells were used as control (Basal, B). Apoptosis was determined via Annexin V assay. Effects of treatment on cell number were expressed as % of control for live, apoptotic and necrotic cells in basal conditions. Experiments were repeated three times. a: p≤0.05 compared to control; b: p≤0.05 compared to 5-FU; c: p≤0.05 compared to 5-FU+L; d: p≤0.05 compared to L.

We investigated the effects of leptin on 5-FU-induced early apoptosis (1-2 days) in PC tumorspheres. The dose-dependent effects of 5-FU on the survival of BxPC-3 (1 day) and MiaPaCa-2 (2 days) cells from tumorspheres were determined via Annexin V assay (Figure 6). The dose 20 μg/ml 5-FU reduced cell survival about 50-60% in BxPC3 (Figure 6A) and MiaPaCa-2 cells (Figure 6B). Thus, 20 μg/ml 5-FU dose was used in future experiments. To determine whether leptin-induced ABCC proteins are involved in the impairment of 5-FU cytotoxicity, PC tumorspheres were treated with 5-FU, leptin, and Probenecid (an inhibitor of ABCC protein function). Leptin increased survival in both cell lines. The addition of DAPT decreased leptin's effects on BxPC-3 cells survival (Figure 6C), but did not influence its effects on MiaPaCa-2 tumorspheres (Figure 6D). 5-FU significantly reduced the number of live cells in PC tumorspheres from both cell lines (Figure 6C and 6D). Leptin decreased the effects of 5-FU on cell survival in PC tumorspheres (Figure 6C and 6D). Remarkably, Probenecid significantly reduced leptin's pro-survival effects, suggesting that leptin-induced expression and increase of ABCC5^+^ and ABCC11^+^ cells play an important role in leptin-mediated impairment of 5-FU cytotoxic effects in PC tumorspheres (Figure 6).

### Leptin decreases 5-FU induced Caspase-3 activation and degradation of PARP

**Figure 7:**
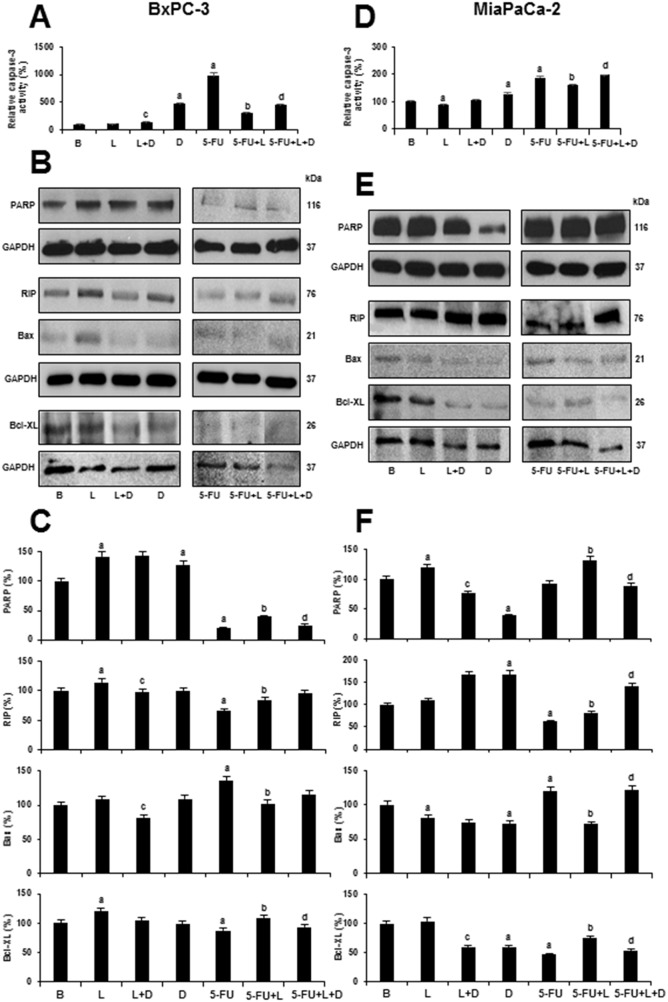
Leptin impairs 5-FU induced Caspase-3 activity and apoptosis- related proteins in 5-FU treated- tumorspheres Caspase-3 activity in BxPC-3 **(A)** and MiaPaCa-2 **(D)** tumorspheres after treatment; Representative western blot results from PARP, RIP, Bax and Bcl-XL expression in BxPC-3 **(B)** and MiaPaCa-2 **(E)** derived tumorspheres; Quantitative determination of PARP, RIP, Bax and Bcl-XL in BxPC-3 **(C)** and MiaPaCa-2 **(F)** tumorspheres after treatment. PC tumorspheres were cultured in 6 wells low-attachment plates in Mammocult medium containing 5-FU (20 μg/ml), leptin (L; 1.2 nM), IONP-LPrA2 (I; 0.0036 pM) and DAPT (D, γ-secretase inhibitor; 20 μM) for 1-2 days. Untreated cells were used as control (Basal, B). Effects of treatment on cell number were expressed as % of control. Experiments were performed three times. a: p≤0.05 compared to control; b: p≤0.05 compared to 5-FU; c: p≤0.05 compared to D; d: p≤0.05 compared to 5-FU+L.

Activation of Caspase-3 leads to the cleavage of a broad spectrum of cellular target proteins, including PARP [poly(ADP-ribose) polymerase] during the induction of apoptosis. PARP is one of the well-known substrates of Caspase-3 and its cleavage is considered a hallmark of apoptosis. PARP cleavage results in formation of two specific fragments: an 89-kDa catalytic fragment and a 24-kDa fragment [39]. When Caspase-3 activity is increased, there is a decrease of uncleaved PARP levels. To investigate the mechanism through which leptin impairs 5-FU cytotoxicity, we examined whether 5-FU and leptin affect Caspase-3 activity using a colorimetric assay and through the detection of uncleaved PARP levels via WB analysis. 5-FU significantly induced Caspase-3 activation in PC tumorspheres from BxPC-3 (Figure 7A) and MiaPaCa-2 (Figure 7D) that was decreased by leptin. Leptin also decreased the basal levels of Caspase-3 activation in MiaPaCa-2 cells (Figure 7D). In contrast, DAPT induced Caspase-3 activity in both cell lines and abrogated leptin's effects in BxPC-3 cells (Figure 7A). Moreover, DAPT plus 5-FU treatment reduced leptin's effects on Caspase-3 activity in both cell lines (Figure 7A and 7D). Consequently, 5-FU decreased the levels of uncleaved PARP in tumorspheres from both cell lines (Figure 7B, 7C and 7E, 7F). However, leptin increased the levels of uncleaved PARP in PC tumorspheres treated with 5-FU (Figure 7B, 7C and 7E, 7F). The inhibition of Notch signaling using DAPT reduced leptin's effects on PARP levels in MiaPaCa-2 tumorspheres. Moreover, DAPT further decreased the levels of uncleaved PARP in both BxPC-3 and MiaPaCa-2 tumorspheres treated with 5-FU and leptin (Figure 7B, 7C and 7E, 7F). Overall, these results suggest that leptin attenuated 5-FU-induced apoptosis in PC tumorspheres. These leptin's effects were Notch dependent.

### Leptin impairs 5-FU's effects on RIP, Bax and Bcl-XL in PC tumorspheres

**Figure 8:**
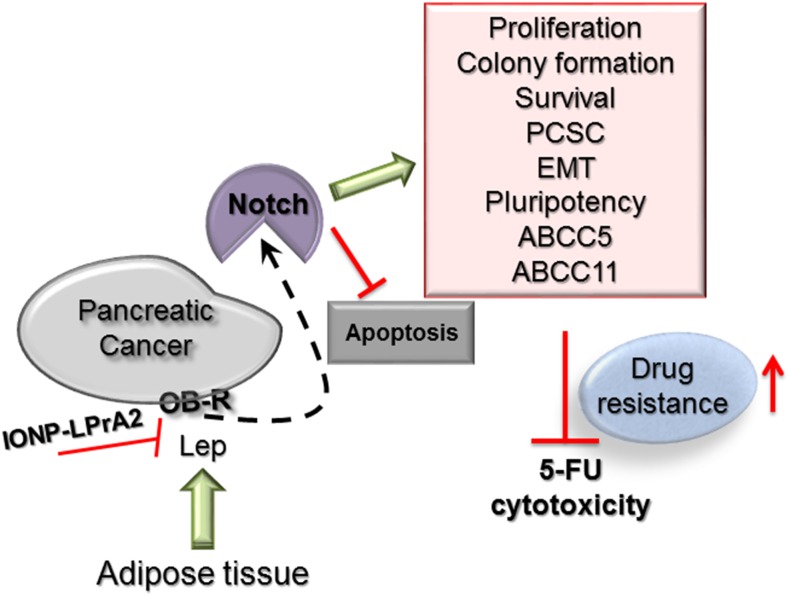
Leptin-Notch signaling axis negatively affects 5-FU cytotoxicity in PC tumorspheres Leptin binding to its receptor (OB-R) expressed by pancreatic cancer tumorspheres induces Notch levels and the number of Notch+ cells that in turn increases proliferation, colony formation, survival, PCSC (CD24+/CD44+/ESA+, c-Met+), EMT (VM+, N-cadherin+), pluripotency (Oct4+, Sox-2+, Nanog+) and ATP-binding cassette proteins (ABCC5+, ABCC11+). Leptin-induced Notch signaling was related to the decrease of 5-FU induced apoptosis (Annexin V+ cells, Caspase 3 activity, Bax, degradation of PARP) and increased levels of anti-apoptotic proteins (Bcl-XL, RIP). Leptin's effects were abrogated by the IONP-LPrA2 leptin signaling inhibitor.

To further gain insight into the mechanism underlying leptin effects on 5-FU-induced apoptosis in PC tumorspheres, WB analysis was used to determine the levels of RIP, Bcl-XL and Bax. 5-FU reduced RIP and Bcl-XL levels in both BxPC-3 (Figure 8A and 8C) and MiaPaCa-2 tumorspheres (Figure 7E and 7F). Additionally, 5-FU increased Bax levels in PC tumorspheres (Figure 7E and 7F). Leptin increased RIP and Bcl-XL basal expression in BxPC-3 tumorspheres (Figure 7E and 7F). Notably, leptin impaired the effects of 5-FU on Bax, Bcl-XL, and RIP levels in both PC tumorsphere types (Figure 7E and 7F). DAPT increased RIP and decreased Bcl-XL basal levels in MiaPaCa-2 tumorspheres (Figure 7F) and decreased the effects of leptin on Bax and Bcl-XL in 5-FU treated PC tumorspheres (Figure 7E and 7F). These results suggest that leptin signaling impairs 5-FU induced apoptosis by decreasing Bax (an activator of apoptosis) and increasing RIP and Bcl-XL (anti-apoptotic proteins). Leptin's effects on Bax and Bcl-XL levels in 5-FU treated tumorspheres were Notch- signaling dependent.

## DISCUSSION

Current epidemiological data show that pandemic obesity is strongly linked to the increased incidence and poor prognosis of several cancers, including PC [40]. A factor that could be involved in these relationships is leptin. Obesity is a modifiable risk factor of PC that is characterized by inflammation and high levels of the adipokine leptin, which was previously found to induce cancer proliferation and expansion of PCSC [23].

Here, we used an *in vitro* model (PC tumorspheres) from two PC cell lines (BxPC-3: less aggressive and MiaPaCa-2: more aggressive) to investigate whether leptin could be an endogenous factor contributing to the reduction of 5-FU cytotoxic effects on PC. Tumorsphere system from PC cells can closely mimic *in vitro* tumor development [41]. Indeed, the formation of 3D spheroids from cancer cells in culture can increase cell resistance to various cancer therapies when compared to monolayers cell culture [42].

We have previously reported that leptin induces Notch expression and signaling in PC cells, which increases tumor progression. Moreover, leptin autocrine and paracrine signaling loop found in PC cells could reinforce its effects on tumor development and PCSC, thus contributing to chemoresistance [23]. The resistance to drug actions is a common characteristic of cancer cells. However, acquired resistance is an additional issue found in cancer patients treated with chemotherapeutics. There are multiple mechanisms involved in the development of chemoresistance, which include the essential actions of drug-efflux proteins (ATP-binding cassette proteins) [43, 44].

One of the most commonly used chemotherapeutic drug for PC is 5-FU. After initial 5-FU treatment, patients commonly develop drug resistance. Recent studies have demonstrated that 5-FU resistance is a complex process, which includes the effects of tumor microenvironment (i.e., desmoplastic reaction of PC dense stroma), highly resistant cells (i.e., PCSC), several factors and molecules that interact with intracellular signaling pathways [7, 45]. Among these signaling pathways, an integrated network involving STAT3, NFkB, AKT and ERK plays important roles in 5-FU resistance [45]. Remarkably, leptin signaling induces the activation of these pathways in normal and cancer cells [46, 47]. It has been shown that leptin can affect EMT, PCSC, and crosstalk to microRNAs (miRNAs) and HDAC to induce PC drug resistance [23, 48–51]. Therefore, leptin is a potential candidate for increasing 5-FU chemoresistance in PC [52, 53].

Reports show that 5-FU, like other chemotherapeutic agents, mainly kills cells that are differentiated and represent the bulk of the tumor, while it spares cells that have stem cell capacity [6, 54]. Oct-4, a PCSC marker, has been previously linked to leptin receptor OB-R expression in cancer stem cells. A statistically significant correlation between the expression of Oct-4 and OB-R across diverse human cancers, including PC, has been found [38]. We have shown that leptin signaling in PC was strongly linked to the increased expression of Notch and ABCB1 drug-efflux protein, cell proliferation, aggressiveness, stem cell expansion and xenograft growth [23]. In the present work, PC cells derived from 5-FU treated tumorspheres were enriched in Notch (Notch1^+^, Notch3^+^ and Notch4^+^), PCSC (CD24^+^CD44^+^ESA^+^ and c-Met^+^), pluripotency (Oct-4^+^, SOX-2^+^, Nanog^+^) and EMT (N-cadherin^+^) markers, which are features associated with highly aggressive PC tumors [55]. Leptin impaired 5-FU chemotherapeutic effects by increasing expression and number of Notch^+^, PCSC^+^, pluripotency^+^ and EMT^+^ PC cells. Hence, 5-FU eliminated the bulk of PC cells, but spared PCSC that were further rescued by leptin.

It has been reported that ABCC5 and ABCC11, rather than ABCB1, play important roles in 5-FU transport and elimination, which could contribute to chemoresistance development [45]. Here, we expanded this notion by demonstrating that 5-FU reduced the expression of ABCC5 and ABCC11 drug efflux proteins. Importantly, leptin, at concentrations typically found in blood of overweight individuals, impaired 5-FU induced effects on PC tumorspheres. Leptin induced ABCC5 and ABCC11 expression and enhanced the proliferation of PC cells expressing these proteins. These leptin's effects gave survival advantages to PC cells forming tumorspheres by reducing 5-FU cytotoxic effects, which also spares ABCC5^+^ and ABCC11^+^ PC cells.

Bcl-2 family of proteins regulate apoptosis by functioning as activators (i.e., Bax) or inhibitors (i.e., Bcl-XL) of the cell death process. RIP (receptor interacting protein) has been reported in TRAIL-induced activation of NF-kB. In addition, the knockdown of RIP sensitized resistant PC cells to TRAIL-induced apoptosis [56]. Here, we found that 5-FU treatment to PC tumorspheres induced apoptosis that decreased cell viability and reduced PC tumorsphere number and size. 5-FU treated PC tumorspheres showed decreased levels of Bcl-XL, RIP and PARP, and higher levels of Bax and Caspase-3 activation. Leptin signaling reduced 5-FU induced-apoptosis, increased cell survival, as well as PARP, Bcl-XL and RIP levels and reduced Caspase-3 activity and Bax levels. Remarkably, leptin signaling increased the number and size of 5-FU treated tumorspheres. The effects of leptin were more evident in the more aggressive MiaPaCa-2 cells.

Present data suggest that the inhibition of Notch activation by DAPT impaired multiple effects of leptin on PC tumorspheres treated with 5-FU. Thus, leptin-induced Notch signaling was involved in leptin's effects on the increased growth and survival, as well as on the decrease of apoptosis of 5-FU treated tumorspheres. A functional leptin-Notch signaling axis was also essential for leptin's rescue of PCSC^+^, pluripotency^+^, EMT^+^ and ABCC5^+^/ABCC11^+^ cells. However, c-Met expression was not dependent of Notch. Moreover, leptin-induced Notch made PC cells more resistant to 5-FU effects.

In conclusion, leptin, at levels typically found in overweight individuals, impaired 5-FU chemotherapeutic effects and gave additional survival advantages to PC tumorspheres by increasing stemness, pluripotency and metastatic potential, and ABCC5 and ABCC11 protein levels. Overall, leptin consistently impaired 5-FU cytotoxicity by increasing proliferation and survival of PC tumorspheres. Remarkably, the effects of leptin on proliferation, apoptosis and chemoresistance on 5-FU treated PC tumorspheres were mainly Notch dependent (see Figure 8). These data reinforce and extent previous reports suggesting a key role of Notch signaling in the development of drug resistance in cancer [57]. Leptin-Notch axis was early found as an essential process for PC development and thus, it could be a novel therapeutic target. Indeed, the inhibition of leptin signaling via nanoparticle-coupled antagonist (IONP-LPrA2) significantly reduced Notch levels, delayed the onset and decreased PC xenograft growth [23]. Thus, leptin-induced Notch's effects on PC tumorspheres treated with 5-FU could contribute to the reduction of its chemotherapeutic potency during PC treatment. These data could be relevant to better understand the multiple mechanisms through which obesity (leptin signaling) could contribute to 5-FU drug resistance developed by PC patients. Moreover, the inhibition of leptin signaling could be a novel target for adjuvant drugs aimed to enhance efficacy of 5-FU, and other chemotherapeutics, which could be especially relevant for overweight and obese PC patients.

## MATERIALS AND METHODS

### Materials

5-FU (Adrucil) was obtained from Selleck Chemicals (Huston, TX). Recombinant human leptin and Caspase-3 colorimetric assay kit was purchased from R&D Systems (Minneapolis, MN). Probenecid, Notch1-FITC, enhanced chemiluminescence (ECL)-WB stripping buffer and protease and phosphatase inhibitor cocktail were purchased from Thermo Fisher Scientific (Rockford, IL). N- [N-(3, 5-difluorophenacetyl)-L-alanyl]-S-phenylglycine t-butyl ester (DAPT), dimethyl sulfoxide (DMSO); GAPDH, Notch1 and poly-ADP ribose polymerase (PARP) polyclonal antibodies were obtained from Sigma (St. Louis, MO). Caspase-3 and Bax polyclonal antibodies were purchased from Cell Signaling (Danvers, MA); RIP monoclonal antibody was from BD Biosciences (San Jose, CA). Bcl-XL monoclonal antibody was from Bethyl Laboratories (Montgomery, TX). CD24-PE, CD44-APC, Oct-4-PE, Notch3-APC, and Notch4-PE antibodies were purchased from Biolegend (San Diego, CA). ESA-FITC antibody, Mammosphere complete medium, 5% heparin and hydrocortisone hemi succinate were from Stem Cell Technologies (Vancouver, BC, Canada). Milli-Mark anti-Nanog-Alexa Fluor 488 was from EMD Millipore (Billerica, MA). Sox-2, ABCC5 and ABCC11 monoclonal antibodies were purchased from Genetex (Irvine, CA). Notch3 and c-Met antibodies were from Abcam (Cambridge, MA). Notch4 antibody was obtained from Santa Cruz Biotechnology (Dallas, TX). Annexin V-FITC, Annexin-V binding buffer and ViaStain PI staining solution for apoptosis were from Nexcelom Bioscience (Lawrence, MA). Fetal bovine serum (FBS) was purchased from Gemini Bio-Products (Sacramento, CA), and penicillin-streptomycin cocktails were from Gibco (Grand Island, NY). Dubelco's Modified Eagle's Medium (DMEM) was from American Type Culture Collection (ATCC, Manassas, VA). Leptin peptide receptor antagonist 2 (LPrA2) bound to iron oxide nanoparticles was synthetized and purified as previously described [58].

### Tumorsphere culture

PC cell lines (BxPC-3, MiaPaCa-2) were obtained from ATCC and cultured in DMEM supplemented with 10% FBS and 1% Penicillin (100 U/ml)/Streptomycin (100 μg/ml) for less than 30 passages. After confluence was reached, cells were collected, washed with PBS, and cultured at clonal density (10–50 × 103 cells/well) in low adherence 6-wells plates containing tumorsphere medium supplemented with heparin and hydrocortisone. Cells were treated for 2-7 days with 5-FU 20 μg/ml, leptin 1.2 nM (corresponding to 20 ng/ml of serum leptin, which is typical found in overweight individuals) [23], IONP-LPrA2 (leptin inhibitor, 0.0036 pM) [58], DAPT 20 μM (an inhibitor of γ-secretase that is required for Notch activation) [23], and Probenecid 2 mmol/ml (ABCC protein inhibitor) [59]. Cells were cultured in humidified atmosphere at 37°C and 5% CO2 for 7 days. Tumorspheres were defined as spheres with diameter greater than 60 μm and were classified as small (60–100 μm), medium (100–200 μm) and large (>200 μm) according to their size. Tumorspheres were counted by number and size using an optical microscope equipped with an eyepiece reticle (Klarmann Rulings, Inc., Litchfield, NH) and were mechanically dissociated in PBS-EDTA and cells were analyzed by flow cytometry.

### Flow cytometry analysis

PC cells were obtained from tumorspheres by mechanically disruption. Then, cells were treated with 1% BSA for 15 minutes at 4°C to block non-specific antibody-binding sites. Then, to assess PCSC markers, cells were incubated with fluorescent monoclonal antibodies for one hour, washed, and fixed using 3.7% formalin. To detect intracellular antigens (Sox-2, Oct-4, Nanog, ABCC5 and ABCC11), cells were permeabilized for 10 minutes using 0.05% Triton X-100 prior to antibody incubation. To analyze the samples by flow cytometry, scatter FSC vs SSC signals were plotted, and gated to distinguish cells from debris. Next, the number of events positive for a specific marker were expressed as percent of total cells. The gates for positive events were set based on unstained control. To analyze the expression of specific proteins by cells derived from PC tumorspheres, positive cells were placed in a histogram within a gate (R6). Histograms were generated for each cell treatment (Basal, Leptin, 5-FU, 5-FU+L), where the median value describes the mean fluorescence intensity and that corresponds to the mean protein expression. To compare the control (basal) or between treatments (i.e., leptin vs leptin+DAPT or leptin vs 5-FU etc.), the corresponding histograms were overlapped. Then, the difference between the medians was calculated and that was defined as delta mean fluorescence intensity (ΔM) [60, 61]. ΔM correlates to relative protein expression in the sample. For the analysis of ΔM for each protein, PC cells (10,000/sample) were acquired using a flow cytometer (Guava system). Results were analyzed using InCyte program from GuavaSoft 3.1.1 (Millipore, Billerica, MA).

### Western Blot

PC cell lysates from cultured tumorspheres were prepared using RIPA buffer containing protease/phosphatase inhibitors. Protein concentration was determined in each sample using Pierce BCA Protein Assay Kit (Thermo Fisher Scientific, Waltham, MA). Thirty to fifty μg of total protein from cell lysates were loaded on 8–15% SDS-polyacrylamide gels for western blot (WB) analysis. After electrophoresis, protein bands were transferred to nitrocellulose membranes (0.45 μm). After blocking for 30 minutes in 5% skim milk-TBST buffer (TBS plus 0.1% Tween 20), the membranes were incubated with primary antibodies overnight at 4°C, followed by incubation with horseradish peroxidase (HRP)-conjugated secondary antibodies and chemiluminescent substrate. Specific antigen expressions were evaluated by capture on X-ray film or using an Image Quant LAS400 system (GE Healthcare, Piscataway, NJ). GAPDH was used as the experimental protein loading control.

### Apoptosis assay

PC tumorspheres were treated with 5-FU (20 μg/ml), leptin (1.2nM), DAPT (20 μM) and IONP-LPrA2 (0.0036 pM) or Probenecid (2 mmol/ml) for 1-2 days. Control cells were treated with DMSO. Annexin V-FITC/PI assay was used to identify and quantify apoptotic and necrotic cells. After treatment, cells were dissociated from tumorspheres and were washed with PBS, resuspended in binding buffer, and stained with Annexin V-FITC and PI (Nexcelom Biosciences, Lawrence, MA) for 15 min at room temperature in the dark. Image cytometry measured FITC/PI fluorescence intensity to differentiate between viable (Annexin V-negative, PI-negative), early apoptotic (Annexin V positive, PI-negative) and late apoptotic or necrotic (Annexin V-positive, PI-positive) cells [62]. Then, the cells were analyzed using a Cellometer Vision CBA (Nexcelom Biosciences).

### Caspase-3 assay

Tumorspheres were cultured for 4 days, then treated for 2 days with 5-FU (20 μg/ml), leptin (1.2 nM), IONP-LPrA2 (0.0036pM), Probenecid (2 mmol/ml) and DAPT (20 μM). Tumorspheres were analyzed for Caspase-3 activity using the Caspase-3 Colorimetric Assay Kit (R&D Systems, Minneapolis, MN) following manufacturer's instructions [63]. In brief, floating tumorspheres were spun and the cell culture media was removed. Then, tumorsphere pellets were lysed in the kit lysis buffer. After that, 50 μl of cell lysates (in triplicate), containing 100 μg of protein were placed onto 96 wells plates and incubated at 37°C for 2 hours with 50 μl of reaction buffer and 5 μl of Caspase-3 (DEVD-pNA) colorimetric substrate. Optical densities were then measured on a microplate reader (λ=405 nm).

### Statistical analysis

All experiments were performed at least three times. Statistical comparisons between treatment groups were made using the student *t*-test and one-way ANOVA. Data are presented as means +/− s.e.m. Values for p≤0.05 were considered statistically significant.
